# Satellites, the All-Seeing Eyes in the Sky: Counting Elephant Seals from Space

**DOI:** 10.1371/journal.pone.0092613

**Published:** 2014-03-20

**Authors:** Clive R. McMahon, Hamish Howe, John van den Hoff, Rachael Alderman, Henk Brolsma, Mark A. Hindell

**Affiliations:** 1 Institute for Marine and Antarctic Studies, University of Tasmania, Hobart, Tasmania, Australia; 2 Australian Antarctic Division, Channel Highway, Kingston, Tasmania, Australia; 3 Threatened Species & Marine Section, Department of Primary Industries, Water and the Environment, Hobart, Tasmania, Australia; Institut Pluridisciplinaire Hubert Curien, France

## Abstract

Regular censuses are fundamental for the management of animal populations but, are logistically challenging for species living in remote regions. The advent of readily accessible, high resolution satellite images of earth mean that it is possible to resolve relatively small (0.6 m) objects, sufficient to discern large animals. To illustrate how these advances can be used to count animals in remote regions, individual elephant seals (*Mirounga leonina*) were counted using satellite imagery. We used an image taken on 10/10/2011 to count elephant seals (n = 1790±306 (95%CL)) on the isthmus of Macquarie Island, an estimate which overlapped with concurrent ground counts (n = 1991). The number of individuals *per* harem estimated using the two approaches were highly correlated, with a slope close to one and the estimated intercept also encompassing zero. This proof of concept opens the way for satellites to be used as a standard censusing technique for inaccessible and cryptically coloured species. Quantifying the population trends of higher order predators provides an especially informative and tractable indicator of ecosystem health.

## Introduction

The population trends of apex predators provide invaluable information on the state of the environment in which animals live because the status of a population is an integrated signal of the state of the lower trophic levels and the environments that sustain them. Determining the bio-physical drivers of such change, and identifying which elements are changing, can be difficult, because long times series are needed to accurately describe population trends and to determine the relationships between population fluctuations and broader environmental changes [1]. These time series rely on regular and accurate censuses which are difficult to maintain, especially for animals that occur on remote oceanic islands or in inaccessible locations such as Antarctica [2], because of logistical constraints in accessing these locations.

Recently launched satellites such as Geo-Eye-1 (panchromatic, 0.5 m resolution and multispectral imagery 1.65 m resolution), WorldView-1 (panchromatic, 0.6 m resolution), WorldView2 (panchromatic, 0.46 m resolution and 8-band multispectral imagery 1.8 m resolution) and QuickBird-2 (2.4 m multispectral, 0.6 m panchromatic) provide easily accessible high resolution images of the Earth's surface. This imagery increasingly provides a vital tool for the collection of information that addresses local to global-scale research and policy priorities [3]. High resolution satellite images have allowed ecologists and wildlife demographers to undertake desktop censuses of the distribution and abundances of animal populations, especially those in remote regions of the globe or those that are sensitive to disturbance [4], [5], [6]. Satellite images have been particularly useful in identifying new seabird nesting habitat [4], [7]–[9], determining rookery size and species composition [10] and quantifying temporal changes in colony size [11]. Most seabird studies are presently restricted to identifying entire colonies [12], not individuals, and therefore need to make several assumptions about animal density and intra-colony distribution to make population estimates. Species that are larger than the current satellite imagery resolution avoid such requirements.

Several studies have used satellite images to count larger species at the individual level [5], [13], including marine mammals, although pinnipeds have proven to be difficult [13]. The sole exception is the Weddell seal (*Leptonochotyes weddellii*) whose dark bodies were highly contrasted against the snow covered Antarctic sea-ice, making these an ideal test for the efficacy of satellite imagery to inform about seal abundance and change over time [5]. Southern elephant seals (*Mirounga leonina*) are the largest pinniped species whose terrestrial breeding sites occur on remote sub-Antarctic islands scattered throughout the Southern Ocean. Elephant seals are a relatively well-studied species and are especially tractable for detecting and recording significant changes in the Antarctic marine ecosystem because they are wide-ranging and therefore integrate environmental signals across ocean basins [14]. While it is possible to quantify their population status through regular breeding season censuses surprisingly little is known on the global population status and trends of this species especially the larger populations that breed on South Georgia and islands on the Kerguelen Plateau (>60% of the global population) [15]. Those islands are logistically extremely difficult to census regularly given their size, remoteness and the treacherous terrain that separates the breeding beaches on these islands. Consequently population information at those locations is sparse, collected infrequently and total island abundance estimates and population trends are often based on smaller sub-areas.

Typically, the number of number of breeding female elephant seals present in mid-October is taken as a reliable index of population size and used to monitor trends [16]. This is conventionally done by through annual ground counts which require considerable logistical investment that is difficult to guarantee from year-to-year. Unlike Weddell seals that are highly contrasted with their breeding substrate, female elephant seals are grey/brown coloured and aggregate in harems established on dark volcanic-sand beaches that offer little contrast, which may make them difficult to distinguish in satellite images.

One of the best-studied southern elephant seal populations breeds on Macquarie Island. About 15% of all females breeding at Macquarie Island congregate in spatially stable harems on the Isthmus study area. Elephant seals found within this study area have been censused by foot on a near-daily basis throughout each breeding season from 1988 onward [17]. Such a committed survey effort within an accessible and well defined area makes the Isthmus an ideal site against which to test the idea that southern elephant seals breeding at very remote, difficult to access locations could be accurately censused from satellite imagery.

This study aimed to use satellite imagery of the Macquarie Island isthmus study area captured during the October breeding season to count the number of female harems, the number of seals *per* harem and the total number of seals in the area. These estimates are then ground-truthed against counts of seals made on the same day. From this study we assess the applicability of the method to census seals observed at terrestrial breeding locations where their detection rates might be poor compared to ice-breeding species.

## Methods

This study was carried out at Macquarie Island under ethics approval from the Australian Antarctic Animal Ethics Committee (ASAC 2265) and the Tasmanian Parks and Wildlife Service.

**Figure 1:**
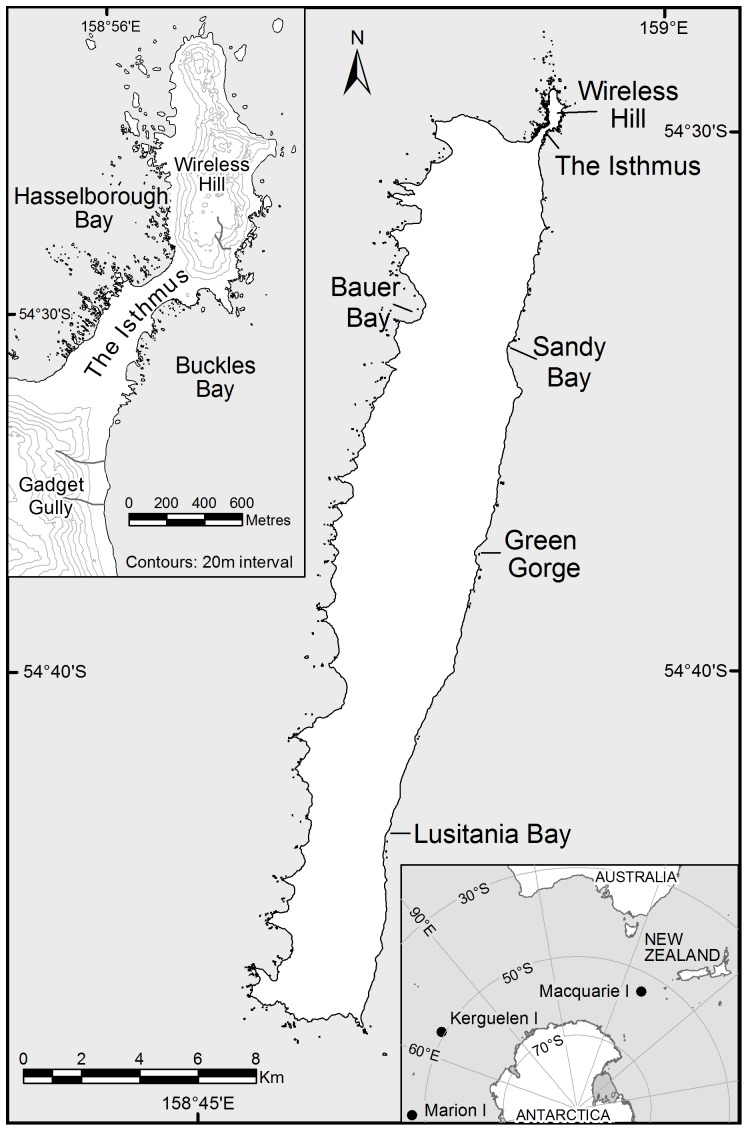
Macquarie Island and the main isthmus study area (upper left panel) and the location of Macquarie Island in the southern Pacific Ocean (lower right panel).

**Figure 2:**
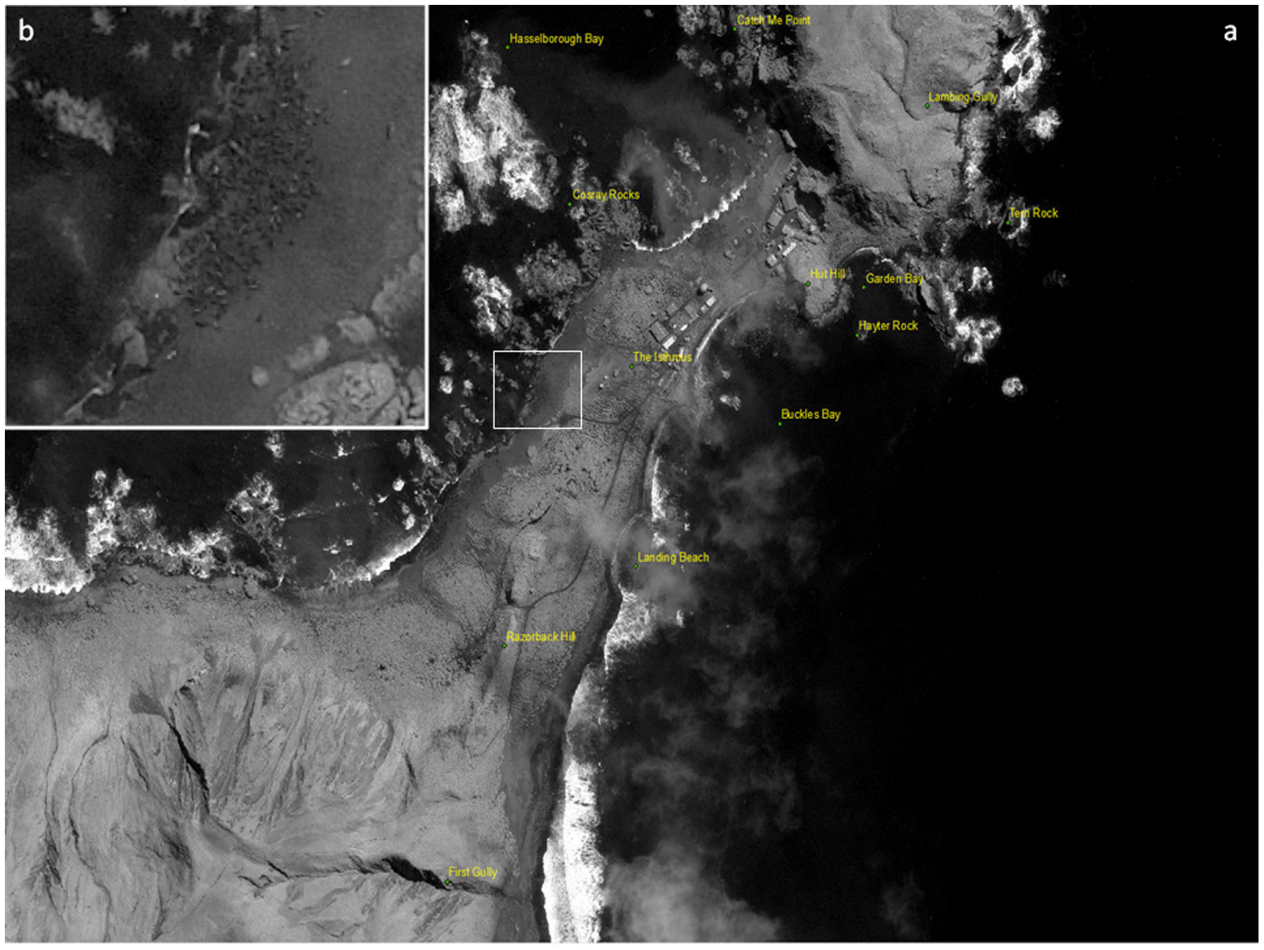
Southern elephant seals - *Mirounga leonina* **, (a) congregate to breed annually in large groups known as harems on remote sub-Antarctic islands.** These harems can be seen from space and individual seals are large enough (2.5 m long) to be counted (b).

To count the number of elephant seals from space we acquired an image of the Macquarie Island isthmus study area (−54.504531°, 158.934330°, Fig. 1) taken on a relatively cloud free day (10/10/2011) by the Geo-Eye satellite. The image is a GeoEye-1 satellite image (DigitalGlobe Catalog ID for the image inumber: 10504100009C3600) captured 10 October 2011 and the off nadir angle is 25.8° (Fig. 2). Image processing was courtesy of the Australian Antarctic Division Data Centre by Angela Bender to produce a pan-sharpened orthorectified image (GE_10Oct2011_ps_orc). The resolution (pixel size) of the pan-sharpened orthorectified image is 0.5 metres. For our study we used the panchromatic Geoeye1 image given this cloud free image coincided with concurrent ground counts on the island. While this was sufficient for our purposes, multispectral sensors such as WorldView2 may be useful for other applications.

A niave observer (HH) searched this image for harems within the study area. We deliberatly used a niave observer to avoid biases introduced by prior knowledge of the island or the distribution and number of harems. Each harem was delineated by a polygon encompassing all animals and assigned a random number. Three counts in random order were made of each harem from a constant virtual height of 300 m and all seals in the harems were counted, using simple image viewing software (Google Earth). The mean of these three counts was taken to represent the number of seals in that harem.

Isolated males and juveniles were excluded from the satellite counts because female counts are the standard way of quantifying population size in elephant seals. New born and nursing pups are approximately 1 m long at this time and coloured black, therefore it is unlikely they would have been counted along with other larger harem seals. No effort was made to distinguish between adult male and female seals within the harem polygons. Including males would not greatly affect counts within the harems because typically there is approximately one male per 50 female seals [18] *i.e.* the total count for the isthmus would be biased by only 40 seals given a total isthmus population of approximately 2000 adult female seals. It is also important to note that there is no diel pattern to elephant seal haulout as the seals remain ashore with their pups for 3 weeks, so time of day that the image was taken relative to the ground count is not a consideration in this study.

Only once the satellite counts had been completed did we compare them to the ground counts. Consequently, there was no prior knowledge of the ground counts at the time of the analyses of the satellite images. Ground counts of individual harems of female seals on the Macquarie Island isthmus were made by two observers. When individual ground counts differed, subsequent counts were done until the final tallies were within 5% [17]. These counts formed part of the regular annual monitoring programme whereby female elephant seals are counted throughout the breeding season at daily to weekly intervals. Fortuitously the ground count made on the 10th October 2011 coincided with acquisition of a cloud-free satellite image.

The satellite counts for each harem were compared to the corresponding ground counts for each harem using linear regression. The satellite counts and the ground counts of seal numbers were taken to be equivalent when the 95% CL of the mean satellite estimate overlapped the ground count.

## Results

Here we demonstrate that despite their cryptic colouration, vital demographic information in the form of population censuses for elephant seals can be collected remotely by satellites, and that the abundance of seals estimated from satellite imagery were an accurate representation of numbers of seals counted on the beach on the same day. The Geo-Eye satellite image contained sufficient resolution to firstly locate all 12 harems on the isthmus study area (Fig. 2). Further, the mean satellite count of elephant seals within the study area was 1790±306 (n = 3 counts; 95% confidence limit) seals, compares well with the ground count of the study area made on the same day: 1991 adult females within 12 harems, which lies within the confidence limits of the satellite estimate.

**Figure 3:**
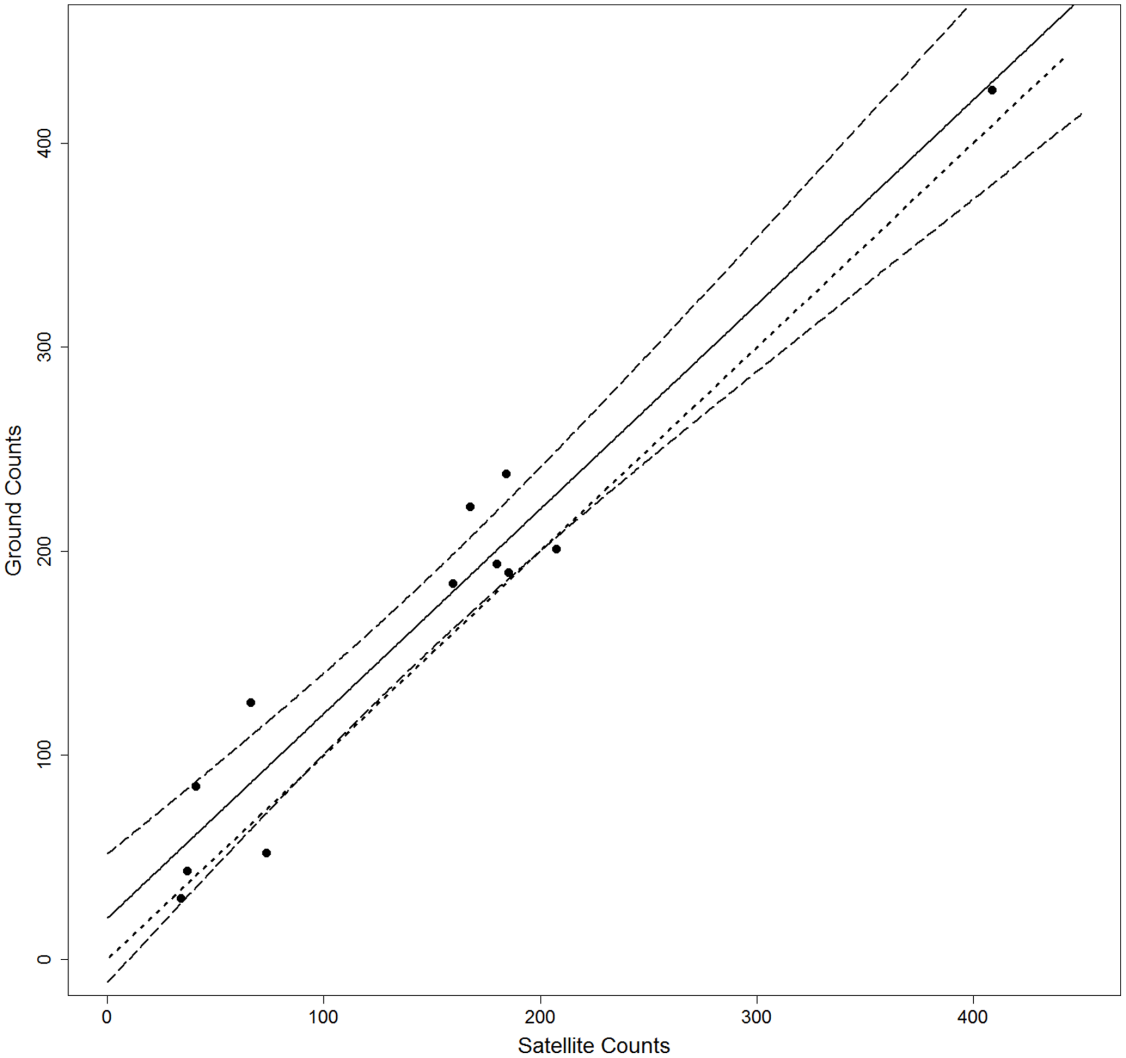
The number of seals counted from space was highly correlated to the actual numbers on beach determined by simultaneous ground counts (*Ground Counts*  = 20.11+1.0003*Satellite counts). The solid line represents the line of best fit, the dashed lines indicated the 95% Confidence limits of that line, and the dotted line is the line of parity (*i.e. Ground Counts*  =  *Satellite counts*).

A linear regression relating the number of seals per harem from satellite imagery and ground counts had a highly significant relationship (F_1,10_ = 107.3, r^2^ = 0.9062, *Ground Counts*  = 22.03+0.946*Satellite counts). The slope of this relationship was 0.964±0.21(95% confidence limit) which encompasses 1.0 and the intercept of the relationship was 22.03±37.79 (95% confidence limit) thereby also encompassing zero. The line of parity (where ground counts equal satellite counts) is also contained within the 95% confidence intervals of the relationship (the dotted line in Fig 3).

## Discussion

We show that individual elephant seals can be reliably and accurately counted from simple, single-spectrum satellite images using manual counting, thereby demonstrating the quick simple and relatively inexpensive utility of the technique. While it was undoubtedly fortuitous that an image was available that was taken on a cloud free day corresponding to a concurrent ground count, this limitation can be resolved given that many of the satellites can be directed to take specific images at specific times, although at some cost to the user. We found no difference between satellite and ground counts for: the total number of harems, the total number of seals on the isthmus, or the number of seals *per* harem, demonstrating that remotely sensed images can reliably be used to census robustly elephant seals on remote sub-Antarctic islands. Earlier work by La Rue [5] demonstrated that individual Weddell seals could be accurately counted from space, but our study is the first to successfully count animals with poor contrast against their background *i.e.* dark bodies on a dark background. This finding greatly broadens the utility of the method, which can potentially be used in many terrestrial and coastal situations.

Despite the congruence of the mean satellite estimates with the ground counts there was nonetheless some disparity between the satellite derived estimates and ground counts. The degree of this disparity *i.e.* the difference between the two types of counts, is important because the power to detect inter-annual variability and abundance trends relies on the precision of annual census data. There are several sources of variance within the estimates. The first is discerning the individual seals. Given that the pixel size of the images is 0.6 m, and that the average length of an adult female seal is 2.4 m and the width is 1.4 m, the seals will be represented in 4–6 pixels, it is unlikely that individual seals were missed. Consequently count variance is most likely due to our in ability to distinguish seals from similar sized rocks and from shading due to sun angle and from animals casting shadows onto adjacent seals in the tightly packed breeding harems [18]. While the harems are tightly packed, elephant seals do not lie on top of one another during the breeding season, and missing seals because they are stacked on top of each other is unlikely.

Resolving the inherent errors of detecting seals in natural settings from satellite images can be relatively easily overcome by (i) increasing the number of replicate counts of the images, thereby reducing the variance around the counts, (ii) by obtaining images on other days during the breeding season when detection parameters may have improved and (iii) by adjusting the image to improve contrast and sharpen edges. This would improve our capacity to distinguish males within the harems and therefore give more accurate counts of females. Alternative methods such as aerial surveys could also improve the accuracy of counts but while aerial surveys give better resolution and hence higher accuracy, such surveys are expensive and often sub-Antarctic islands are not within range of survey aircraft.

Our findings illustrate the general utility of using satellite acquired census information to accurately enumerate the numbers of individual large-bodied animals in the wild. While our findings are a manifestly useful tool for counting elephant seals at remote and rarely visited islands such as Heard Island and the remote beaches of South Georgia which contain about 62% of the World's elephant seals population for which there are no contemporary counts. This techniques of counting seals using satellites can be extended to other Sothern Ocean pinnipeds such as pack-ice seals, other marine mammals such as whales or large terrestrial animals such as zebra, camels, elephants, bison and savannah ungulates that occur in open terrain [19], [20]. This has the potential to revolutionize how animals are censused at a time when robust demographic information including longitudinal count series to quantify population trends and growth rates are sorely needed, especially in the light of the current biodiversity crisis [21]–[22].

## References

[1] McMahon CR, Bester MN, Hindell MA, Brook BW, Bradshaw CJA (2009) Shifting trends: detecting environmentally mediated regulation in long-lived marine vertebrates using time-series data Oecologia. 159: 69–82. 10.1007/s00442-008-1205-9 18987892

[2] Schofield O, Ducklow HW, Martinson DG, Meredith MP, Moline MA, et al (2010) How do polar marine ecosystems respond to rapid climate change? Science 328: 1520–1523. 20558708 10.1126/science.1185779

[3] Horning N, Robinson J, Sterling E, Turner W, Spector S (2010) Remote sensing for ecology and conservation: Oxford University Press. 448 p.

[4] Hughes BJ, Martin GR, Reynolds SJ (2011) The use of Google Earth (TM) satellite imagery to detect the nests of masked boobies Sula dactylatra. Wildl Biol 17: 210–216.

[5] LaRue MA, Rotella JJ, Garrott RA, Siniff DB, Ainley DG, et al (2011) Satellite imagery can be used to detect variation in abundance of Weddell seals (Leptonychotes weddellii) in Erebus Bay, Antarctica. Polar Biol 34: 1727–1737.

[6] Platonov NG, Mordvintsev IN, Rozhnov VV (2013) The possibility of using high resolution satellite images for detection of marine mammals. Biol Bull 40: 197–205. 23789427

[7] Fretwell PT, Trathan PN (2009) Penguins from space: faecal stains reveal the location of emperor penguin colonies. Glob Ecol Biogeogr 18: 543–552.

[8] Fretwell PT, LaRue MA, Morin P, Kooyman GL, Wienecke B, et al. (2012) An Emperor Penguin Population Estimate: The First Global, Synoptic Survey of a Species from Space. Plos One 7.. 10.1371/journal.pone.0033751 PMC3325796 22514609

[9] Trathan PN, Fretwell PT, Stonehouse B (2011) First Recorded Loss of an Emperor Penguin Colony in the Recent Period of Antarctic Regional Warming: Implications for Other Colonies. Plos One 6. 10.1371/journal.pone.0014738 PMC3046112 21386883

[10] Lynch HJ, White R, Black AD, Naveen R (2012) Detection, differentiation, and abundance estimation of penguin species by high-resolution satellite imagery. Polar Biol 35: 963–968.

[11] Naveen R, Lynch HJ, Forrest S, Mueller T, Polito M (2012) First direct, site-wide penguin survey at Deception Island, Antarctica, suggests significant declines in breeding chinstrap penguins. Polar Biol 35: 1879–1888.

[12] LaRue MA, Ainley DG, Swanson M, Dugger KM, Lyver POB, et al (2013) Climate Change Winners: Receding Ice Fields Facilitate Colony Expansion and Altered Dynamics in an Adélie Penguin Metapopulation. Plos One 8: e60568. 23573267 10.1371/journal.pone.0060568 PMC3616090

[13] Laliberte AS, Ripple WJ (2003) Automated wildlife counts from remotely sensed imagery. Wildl Soc Bull 31: 362–371.

[14] Hindell MA, Bradshaw CJA, Guinet C, Harcourt RG (2003) Ecosystem monitoring and modelling: can marine mammals signal or predict change? In: Gales N, Hindell MA, Kirkwood R, editors. Marine mammals and humans: towards a sustainable balance. Melbourne: CSIRO Publishing.

[15] McMahon CR, Bester MN, Burton HR, Hindell MA, Bradshaw CJA (2005) Population status, trends and a re-examination of the hypotheses explaining the recent declines of the southern elephant seal *Mirounga leonina* . Mamm Rev 35: 82–100.

[16] Hindell MA, Burton HR (1987) Past and present status of the southern elephant seal (*Mirounga leonina*) at Macquarie Island. J Zool (Lond) 231: 365–380.

[17] Van den Hoff J, Burton HR, Raymond B (2007) The population trend of southern elephant seals (Mirounga leonina L.) at Macquarie Island (1952–2004). Polar Biol 30: 1275–1283.

[18] McMahon CR, Bradshaw CJA (2004) Harem choice and breeding experience of female southern elephant seals influence offspring survival. Behav Ecol Sociobiol 55: 349–362.

[19] Gutro R (2005) Satellite Data to Track Wildlife: Elephants in Space. Available: http://www.nasa.gov/vision/earth/lookingatearth/elephants_space.html. Accessed 2013 October 1.

[20] Yang Z (2012) Evaluating high resolution GeoEye-1 satellite imagery for mapping wildlife in open savannahs. Enschede, The Netherlands University of Twente 1–61 p.

[21] Reich PB, Tilman D, Isbell F, Mueller K, Hobbie SE, et al (2012) Impacts of biodiversity loss escalate through time as redundancy fades. Science 336: 589–592. 22556253 10.1126/science.1217909

[22] Hooper DU, Adair EC, Cardinale BJ, Byrnes JEK, Hungate BA, et al (2012) A global synthesis reveals biodiversity loss as a major driver of ecosystem change. Nature 486: 105–108. 22678289 10.1038/nature11118

